# Endovascular treatment of traumatic thoracic aortic injuries in patients with normal anatomy and anatomical variants: safety, efficacy and long-term follow-up

**DOI:** 10.1007/s11547-026-02173-5

**Published:** 2026-01-27

**Authors:** Federico Fontana, Filippo Piacentino, Federica Grimoldi, Andrea Coppola, Edoardo Macchi, Maria Cristina Cervarolo, Marco Franchin, Anna Maria Ierardi, Chiara Floridi, Gianpaolo Carrafiello, Andrea Giovagnoni, Massimo Venturini

**Affiliations:** 1 Diagnostic and Interventional Radiology Unit, Circolo Hospital, ASST Sette Laghi, 21100 Varese, Italy; 2 Department of Medicine and Technology Innovation, Insubria University, 21100 Varese, Italy; 3 Department of Science and High Technology, University of Insubria, 22100 Como, Italy; 4 Vascular Surgery Unit, Circolo Hospital, ASST Sette Laghi, 21100 Varese, Italy; 5 Department of Diagnostic and Interventional Radiology, Foundation IRCCS Cà Granda-Ospedale Maggiore Policlinico, Via Francesco Sforza 35, 20122 Milan, Italy; 6 Department of Clinical, Special and Dental Sciences, University Politecnica Delle Marche, 60126 Ancona, Italy; 7 Faculty of Health Science, Università Degli Studi Di Milano, Via Festa del Perdono 7, 20122 Milan, Italy

**Keywords:** TEVAR, Traumatic thoracic aortic injury, Endovascular, Long-term follow-up, Anatomical variants, MRI, Left subclavian artery

## Abstract

**Purpose:**

To evaluate the safety and the efficacy of thoracic endovascular aortic repair (TEVAR) in patients with traumatic thoracic aortic injuries (TTAIs), analyzing the mid-long-term outcomes.

**Materials and methods:**

In this retrospective and monocentric study, 20 patients (46 ± 17.0 years old; mean ± SD) underwent TEVAR for TTAI from February 2012 to April 2023. All patients were subjected to computed tomography angiography (CTA) before discharge; afterward, the follow-up was set up by CTA or magnetic resonance imaging (MRI). Technical success, clinical success, safety, and mid-long-term follow-up were evaluated.

**Results:**

Both technical and clinical success were 100%. No procedure-related death was reported. Safety was 95%. In four (20%) patients the left subclavian artery (LSA) was covered; in one out of these four (25%), revascularization was necessary through carotid-subclavian bypass. In a patient with an anatomic variant of aberrant right subclavian artery (ARSA) a stent placement was required. The follow-up’s median duration was 17 ± 79.5 months (median ± IQR) and in three cases there were minimal complications: a stable type IA endoleak (EL) < 1 cm, a minimal irregularity of device’s links, and a millimeter bird beak sign.

**Conclusions:**

TEVAR for TTAI was found to be safe (3 minimal complications) and effective both in the short and mid-long term. Patients’ adherence to follow-up and contrast-induced kidney damage remains a challenge, but the use of MRI may be a valid alternative, avoiding ionizing radiation and the use of iodinated contrast media.

## Introduction

Traumatic thoracic aortic injuries (TTAIs) are rare conditions (annual incidence of 1,5–2%) [1, 2], but associated with high mortality [3–5]: about 77–85% of victims do not reach the hospital and mortality is 95% if TTAIs are not treated [1, 4, 6, 7]. The most frequent cause of aortic traumas are motor vehicle collisions (about 80%) [3, 6, 8–11], followed by precipitations and crushing traumas [5, 8–10]. Males are more affected than females, with a mean age from 35 to 40 years old [3, 10]. The most involved aortic segment in traumatic injury is the isthmus region (zone 3) [3, 5, 6, 9, 10, 12].

TTAIs can be distinguished into four categories, according to Azizzadeh et al. [13] classification: grade I, intimal tear; grade II, intramural hematoma; grade III, pseudoaneurysm; grade IV, rupture. This classification is also widely used to set the correct treatment course [3].

Clinical presentation is not diriment to assess correct diagnosis: hemodynamic instability is found in less than half of patients, who are often paucisymptomatic (chest pain, interscapular pain, or abdominal pain is usually referred) or asymptomatic.

So, it is crucial to suspect an aortic lesion in every high kinetic energy trauma (especially in thoracic ones); moreover, is decisive to comprehend the event’s dynamic particularly in high energy trauma [5, 6, 8–10, 14].

The first-level exam may be chest radiography [9, 10], but it remains poorly sensible and specific [3], causing an elevated number of false negatives [5, 15]. The diagnostic gold standard is computed tomography angiography (CTA) [3, 5, 9, 12], capable of recognizing, in a short time and with very high sensitivity and specificity, aortic lesions and their site; allowing a rapid treatment’choice [3, 9, 12].

Nowadays TTAIs main treatment is represented by thoracic endovascular aortic repair (TEVAR) [3, 15], a method born in the early 90 s [16, 17] and indicated by Society of Vascular Surgery guidelines since 2011 [7] (confirmed by the latest 2017 guidelines) [3, 15]. From the moment of its introduction in 1994 (Dake et al.) [16] to today, the use of TEVAR is significantly increased, at the expense of classical open repair, which is associated with high complications and mortality [4, 18].

The long-term effectiveness of TEVAR is yet to be defined [4, 5, 9].

Several issues emerged, including the size of prostheses to be placed (usually designed for senile and tendentially dilated aortas) [4], the hemodynamic changes caused by the device [19], and the possible growth of the aorta (especially in younger patients) [20].

In the literature are available numerous studies reporting the safety and efficacy of TEVAR in acute and chronic aortic disease, while to our knowledge TEVAR for TTAIs is less reported. In addition, the follow-up is poor. This retrospective, single-center study aims to define the safety and efficacy of TEVAR in TTAIs, also evaluating its mid-long-term follow-up and comparing our findings with the current literature.

## Material and methods

### Population

This retrospective, single-center study enrolled a cohort of consecutive patients who underwent TEVAR for TTAIs from February 2012 to April 2023.

Patients with an acute thoracic trauma, underwent clinical evaluation and whole-body CTA; once the aortic lesion was diagnosed, lesions type (II, III, IV grade) [13] and any anatomical variant, a TEVAR procedure was scheduled following a multidisciplinary team evaluation of the case, including emergency physicians, anesthesiologists, general surgeons, interventional radiologsts, and vascular surgeons.

The endoprosthesis was chosen based on the CTA findings and diagnostic angiography to ensure complete coverage of the aortic lesion and gain an adequate distal and proximal landing.

### TEVAR procedure

All procedures were performed in the operating room by a team consisting of interventional radiologists, vascular surgeons, and anesthesiologists with a portable C-arm angiography system (Vision RFD; Ziehm, Nuremberg, Germany; BV 300 C-Arm; Philips Medical System, Best, the Netherlands). Patients underwent general anesthesia, with cardiorespiratory monitoring and antibiotic prophylaxis (cefazolin 2 gr or vancomycin 1 gr).

Peripheral vascular accesses (right and left common femoral arteries) were surgically isolated, according to patient anatomy and procedure necessity; in some patients, a humeral access was surgically isolated or percutaneous gained. From the femoral access, through a 5 French (F) introducer, a 0.035’’ hydrophilic guide was advanced in the ascending aorta and then exchanged with a stiff guide wire (Lunderquist Extra-Stiff, Cook Medical, Bloomington, Indiana, USA). A 22 F introducer (Dryseal, W. L. Gore & Associates, Inc., Newark, Delaware, United States) was brought into the abdominal aorta. A co-axial pigtail-shaped angiographic catheter (Supertorque MB, Cordis, Hialeah, Florida, United States) was advanced to the aortic valve. An aortography was performed to confirm the CTA findings using from 24 to 36 °C of contrast media (Visipaque 320 mg I/ml, GE HealthCare, Chicago, Illinois; about 200 ml to patient) and was used to guide the future step of the procedure. The stent grafts were chosen by oversizing approximately 10–20% relative to the native thoracic aortic diameters, and with an appropriate length to cover the aortic lesion completely. The prostheses used were the Gore TAG Conformable (C-TAG; W. L. Gore & Associates, Inc., Newark, Delaware, United States) and the Medtronic Valiant Captivia (Medtronic, Dublin, Ireland). Once the aortic lesion is identified and the endograft chosen, the stent-graft was advanced on the stiff guidewire and then released to cover the lesion.

In cases where a proximal landing zone with a length of less than 1.5–2 cm was present, the left subclavian artery ostium had to be covered and obliterated. After the endoprosthesis was placed, a second aortography was performed to assess the correct stent-graft positioning and exclude complications.

### Outcomes and Follow-up

Technical success and clinical success were evaluated. Technical success was based on the correct placement and patency of the aortic endoprostheses at the end of the procedure. Clinical success was based on patient survival and correct positioning and patency of the device at 30 days.

Complications were reported as defined by the CIRSE Quality Assurance Document and Standards for Classification of Complications [21].

All patients underwent postoperative CTA before discharge and were directed to follow up by CTA control at 1, 6, and 12 months and annually or biannually thereafter; alternatively, MRI was used in case of contraindications to CTA (kidney failure or iodinated contrast medium allergy). During follow-up, aortic size, prostheses characteristics, and possible presence of complications were evaluated. Patients were contacted by telephone to ascertain the continuation of follow-up and their health status.

### Statistics analysis

In this observational study, only descriptive statistics were produced. Numbers and percentages are presented for categorical variables, and mean and standard deviation (SD) are presented for normally distributed variables. Kolmogorov–Smirnov test was used to assess normality.

## Results

### Population

Twenty patients were recruited, including 15 men and 5 women, with a mean age of 46 ± 17.0 years old (mean ± SD; range: 19–79 years old), undergoing TEVAR for TTAIs. The general characteristics of the study population are shown in Table 1 and Table 2. Ten (50%) patients had no pathologies at the time of the trauma; considering the remaining 10 patients, seven (35%) had a metabolic syndrome, including hypertension and diabetes mellitus. The most frequent cause of injury was motor vehicle collisions (15 cases, 75%), followed by precipitation (4 cases, 20%), and crushing (one case, 5%). Six (30%) patients presented with unstable hemodynamics; in the 14 hemodynamically stable subjects, five (25%) arrived intubated, 2 (10%) were completely asymptomatic, and the remaining were clinically paucisymptomatic.

**Table 1:** Summary table

	No	(%)	Mean ± SD	Range
Age			46 ± 17.0	19–79
Sex				
M	15	75		
F	5	25		
Comorbidity				
CV	7	35		
Not CV	2	10		
Etiology				
Motor vehicle collision	15	75		
Precipitation	4	20		
Crushing	1	5		
Clinical presentation				
Thoracic-abdominal pain	7	35		
Unstable hemodynamics	6	30		
OTI	5	25		
Asymptomatic	2	10		
Paraplegia	1	5		
TAI grade				
II	1	5		
III	16	80		
IV	3	15		
Anatomic variants				
Bovine trunk	3	15		
ARSA	1	5		
Right-sided aortic arch	1	5		
Associated lesions				
Thoracic	17	85		
Limb fractures	14	70		
Abdominal	9	45		
Head	7	35		
Access				
R CIA	1	5		
R EIA	1	5		
Bilateral CFA	1	5		
L CFA	6	30		
R CFA	11	55		
HA	3	15		
Aortic diameter (mm)			23.8 ± 2.7	20–31
Endoprostheses				
Gore C-TAG	19	95		
Medtronic Valiant	1	5		
LSA coverage	4	20		
Technical success	20	100		
Peri-procedural complications	3	15		
Clinical success	20	100		
Follow-up complications	3	15		
Follow-up (months)			41.35 ± 44.23	1–127
1	20	100		
12	10	50		
36	8	40		
> 48	5	25		

*SD* Standard Deviation, *CV* cardiovascular (pathologies related to metabolic syndrome), *OTI* oro-tracheal intubation, *ARSA* aberrant right subclavian artery, *R* right, *L* Left, *CIA* common iliac artery, *EIA* external iliac artery, *CFA* common femoral artery, *HA* humeral artery, *LSA* left subclavian artery

**Table 2:** Summary table of the presented cases

	Age, Sex	Etiology	TAI grade	Anatomic variants	LSA coverage	Complications	Follow-up (months)
#01	28, F	MVC	III	Bovine trunk	No	/	36
#02	44, M	MVC	IV	/	No	/	1
#03	63, M	MVC	III	ARSA	Yes (Periscope technique)	ARSA stent stenosis	108
#04	56, M	Precipitation	III	Right-sided aortic arch	No	/	107
#05	55, F	MVC	III	/	No	Left upper extremity ischemia	15
#06	19, M	MVC	III	/	No	/	102
#07	27, M	MVC	II	Bovine trunk	No	/	94
#08	42, M	MVC	III	/	No	/	74
#09	78, F	MVC	IV	/	No	/	4
#10	47, M	MVC	III	/	No	/	79
#11	20, F	MVC	III	/	No	/	4
#12	79, M	Precipitation	III	/	Yes (Coils)	Misplacement Amplatzer in CCA	1
#13	58, F	Precipitation	IV	/	No	/	1
#14	54, M	Precipitation	III	/	No	/	127
#15	55, M	MVC	III	/	Yes (Amplatzer Vascular Plug)	/	31
#16	21, M	MVC	III	/	No	/	19
#17	42, M	MVC	III	/	No	/	12
#18	46, M	Crushing	III	/	Yes (Surgical)	/	10
#19	36, M	MVC	III	Bovine trunk	No	/	1
#20	51, M	MVC	III	/	No	/	1

*ARSA* aberrant right subclavian artery, *CCA* common carotid artery, *LSA* left subclavian artery, *MVC* motor vehicle collision

**Fig. 1:**
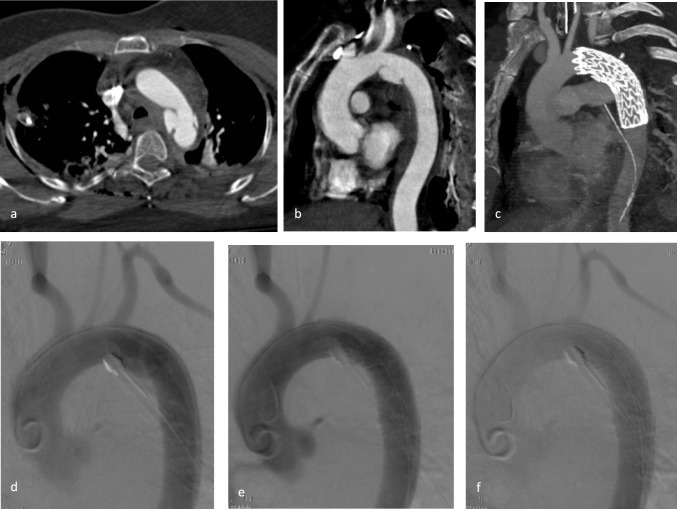
Grade IV aortic isthmic lesion treated with Gore C-TAG 26 × 100 mm. **a b** Preoperative axial and para-coronal CTA and **d** aortography; **c** postoperative para-coronal CTA and **e f** aortography: correct positioning and patency of endoprostheses, no complications; LSA dulling from retrograde revascularization

**Fig. 2:**
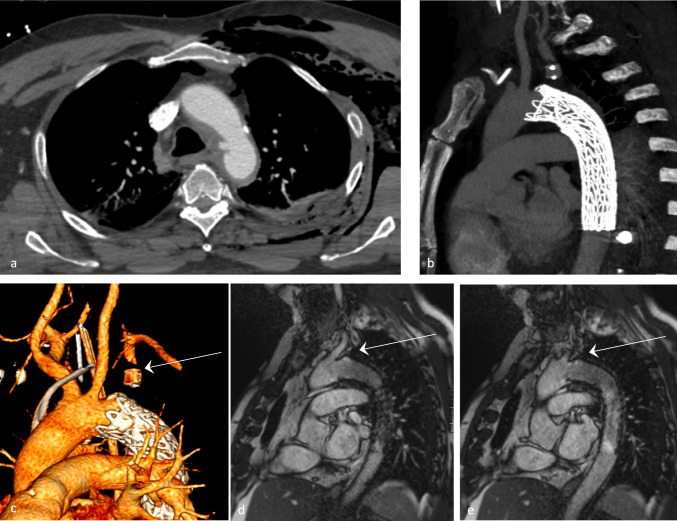
Grade III aortic isthmic lesion treated with Gore C-TAG 34 × 150 mm with coverage and exclusion of LSA (Amplatzer Vascular Plug 12 mm, arrow). **a** Preoperative CTA; **b** postoperative CTA and **c** volume rendering reconstruction; **d e** 31-months follow-up MRI: no device-related complications

**Fig. 3:**
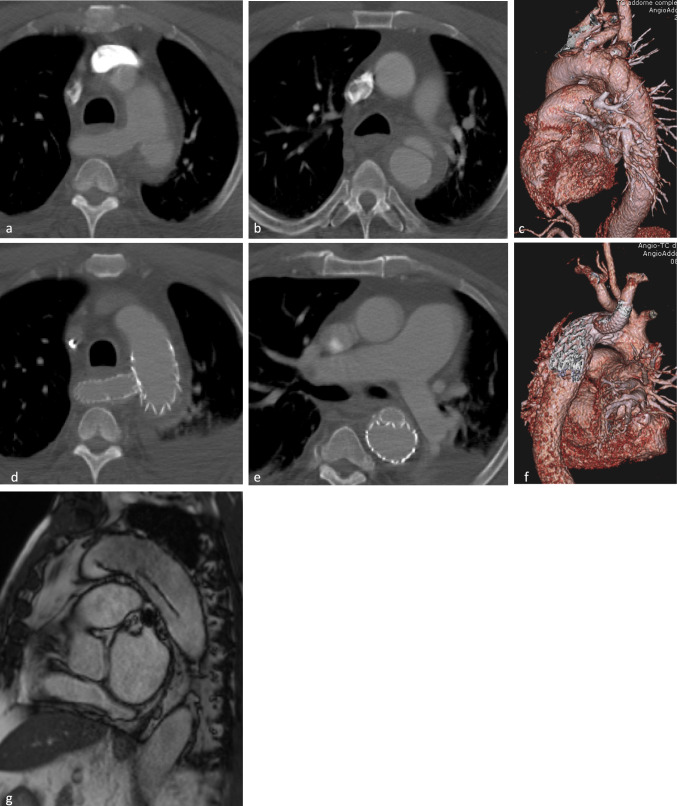
Grade III aortic isthmic lesion in ARSA anatomical variant treated with Gore C-TAG 36 × 150 mm and stent (Viabahn 13 × 100 mm) in ARSA, placed according to periscope technique. **a b** Preoperative CTA and **c** volume rendering (VR) reconstruction; **d e** postoperative CTA and **f** VR: correct placement and patency of aortic endoprotestheses; **g** 108-months follow-up MRI: no prostheses- and stent- related complications

**Fig. 4:**
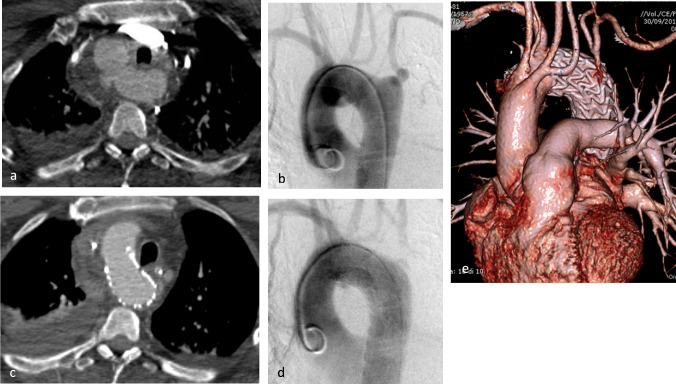
Grade III aortic isthmic lesion in Right-sided aortic arch anatomic variant treated with Gore C-TAG 31 × 150 mm. **a** Preoperative CTA and **b** aortography; **c** postoperative CTA and **d** aortography and **f** volume rendering: correct positioning and patency of endoprostheses, no complications

The mean aortic diameter was 23.8 ± 2.7 mm. All patients presented with the aortic lesion in zone 3 (Figs. 1, 2): out of them, sixteen (80%) subjects had a grade III lesion, 3 (15%) grade IV, and 1 (5%) grade II. Anatomical variants were identified in five (25%) cases (Fig. 3, 4). Also, all patients had other injuries in association with the aortic trauma: in only 2 (10%) cases thoracic trauma occurred isolated.

### Procedure

Peripheral vascular accesses were surgically isolated: eleven (55%) right common femoral artery, 6 (30%) left common femoral artery, 1 (5%) femoral artery bilaterally, 1 (5%) right external iliac artery, and 1 (5%) right common iliac artery; in 3 (15%) cases a humeral artery access also had to be used (1 right and 2 left).

The prostheses used were the Gore C-TAG in 19 (95%) patients, and the Medtronic Valiant in one (5%) patient only. In all patients, only one prosthetic module was placed. The prostheses placed had a mean diameter of 26.8 ± 4.6 mm (mean ± SD; range: 21–36 mm) and a length of 111.5 ± 18.4 mm (mean ± SD; range: 100–150 mm).

The ostium of LSA has been excluded in four (20%) cases: in one case with coils (Concerto Helix, Medtronic, Dublin, Ireland), in two cases with Amplatzer vascular plug (Abbott, Chicago, Illinois, United States) (12 e 16 mm) (Fig. 2), and by surgical ligation in the last case.

In a patient with ARSA, the deployment through humeral access of an 13 mm × 100 mm covered stent (Viabahn, W. L. Gore & Associates, Inc., Newark, Delaware, United States) with the periscope technique (from the ARSA origin to the distal margin of the aortic graft) was required (Fig. 3).

### Outcomes

Technical success was achieved in all cases (100%, Table 1): no patient died in the operating theater; all patients were stable and with properly placed and patent grafts.

We reported 3 (15%) periprocedural complications: in one case left upper extremity ischemia occurred, 15 min after the procedure, which required a Fogarty’s thrombectomy of the humeral-ulnar-radial axis with complete resolution (CIRSE Classification System for Complications Grade I). In a patient with ARSA, stent stenosis was found on XI postoperative day, which was treated with angioplasty (CIRSE Grade III). In the third case, there was a misplacement of a 16 mm Amplatzer in LACC, which was promptly removed by a 12 mm Hooker-type catheter; subsequently, the ostium of LSA was correctly obliterated and revascularization of the subclavian axis was not necessary. A brain CT after the procedure was negative and the patient reported no sequelae (CIRSE Grade I). Thus, the safety of the procedure was 95%.

All patients were alive and the endoprostheses were patent and correctly placed in all cases at 30 days after the procedure. Clinical success was achieved in 100% of the cases, Table 1 and 2). One patient died not from complications related to the procedure but from cardiovascular arrest 41 days after the procedure.

### Follow-up

Follow-up was performed by CTA (17 patients, 85%) or MRI (3 patients, 15%). The follow-up median duration was 17 ± 79.5 months (median ± IQR; range: 1–127 months), and in nine (45%) cases it was also performed by phone call.

During follow-up, the following emerged: (1) a type IA EL, which, however, appears to be stable (< 1 cm, at 43 months); (2) minimal prosthetic mesh irregularity at the distal third of the device (at 36 months); and (3) a millimeter bird beak sign (at 1 month).

At the last follow-up (including the telephone survey) all but one patient, reported well-being and confirmed that they had returned to activities of daily living. Only one patient did not return to daily living due to trauma-related complications (medullary transection and consequent paraplegia).

## Discussion

Endovascular management of aortic and arterial lesions, less burdened by morbidity and mortality than surgery, is generally considered the first therapeutic option [3, 5, 7, 11, 12, 15, 17, 19, 22–26]. From the earliest studies available in the literature about TTAIs, it became apparent that TEVAR avoids some of the typical disadvantages of the open approach, such as single-lung ventilation, aortic clamping, and cardiopulmonary bypass; it also reduces surgical time and blood loss [5]. TEVAR is generally associated with fewer complications without excluding their possible occurrence [4], including death, paraplegia, renal failure, transfusions, reinterventions for bleeding, cardiac complications, pneumonia, and length of recovery [3, 10]. In the literature, there are no randomized clinical trials available that directly compare open repair with endovascular technique, given the clear superiority of TEVAR in terms of reduction of mortality and complications; consequently, it is necessary to compare the two approaches through systematic reviews or retrospective trials [3, 4, 7, 27–30] (Table 3). United States Food and Drug Administration approved the use of TEVAR for TTAIs in 2012 [3], an indication confirmed in 2015 by EAST through a meta-analysis [7], and is currently the SVS-recommended approach in patients with favorable anatomy (2017 guidelines, GRADE IC) [15].

**Table 3:** TEVAR versus Open Repair (OR) for TTAIs: literature review

Author	Journal	Year of publication	Study type	No. of patients	Principal results
Buz et al.[29]		2008	Retrospective	35 (OR)/39 (TEVAR)	More complications and mortality in the OR group
	Eur J Cardiothorac Surg				TEVAR free of peri-procedural complications
					TEVAR is better in terms of early outcomes
Lee et al. [4]	Journal of Vascular Surgery	2011	Systemic Review	1476 (OR)/699 (TEVAR)/3574 (nonsurgical treatment)	TEVAR is associated with less mortality and less incidence of peri-procedural complications compared to OR
Cheng et al. [30]	JAMA Network Open	2019	Retrospective	154 (OR)/133 (TEVAR)	Better long-term outcome of TEVAR compared with OR
Elkbuli et al. [27]	Journal of Surgical Research	2020	Retrospective	103 (OR)/172 (TEVAR)	TEVAR is much superior to OR in terms of mortality

To date, endovascular repair represents the best option for TTAIs [27, 31–33], as confirmed by our study. Open Repair is indicated in cases ineligible for endovascular treatment (mainly due to unfavorable anatomy, including the presence of iliofemoral axis disease or diameters of these vessels less than 7 mm) or in cases where TEVAR fails, requiring open conversion (rate of about 4%) [3, 10].

Preoperative planning based on CTA is crucial for choosing the correct endoprosthesis. The most important and challenging measure is the proximal landing zone: there may be anatomical variants, or the length may be under 20 mm. In these cases, LSA coverage may be indicated [3, 5, 31, 34–36]; it is reported that in trauma LSA coverage occurs in more than half of the cases [37], whereas, in our case series it was needed in only 20% of patients. There are risks related to LSA coverage [3, 37]: in 2022, Grigorian et al. [37] showed that these complications would appear to be greater in patients performing a TEVAR with LSA coverage for TTAIs, compared with those who did not undergo trauma; in our patients who underwent LSA coverage, no one reported clinical complications. If the right vertebral artery is hypoplastic or atretic, if there is an anatomic alteration of the polygon of Willis, or in the case of aortocoronary bypass with internal mammary artery, should consider LSA revascularization through traditional surgery techniques (such as transposition of LSA or carotid-subclavian bypass) or by endovascular techniques (such as chimney technique or fenestrated graft) [4, 15, 36, 37].

Preoperative planning is also important to identify the presence of any anatomic variants; these are not uncommon conditions: a review published by our group [26] found that anatomic variants were present in 15–34% of cases; in particular, aberrant right subclavian artery was described in 0.4–1.2% and right-sided aortic arch in 0.1–0.2%. In these cases, the application of TEVAR in emergencies remains controversial. In our cohort, there were five patients with anatomic variants: one (5%) aberrant right subclavian artery, one (5%) right-sided aortic arch, and three (15%) bovine trunk; all of them were treated with TEVAR, and all prostheses are still correctly positioned and patent at follow-up.

The most frequently encountered complication following TEVAR is type I endoleak [3]. Other complications include stroke and spinal cord ischemia [4], access site complications (such as infection and bleeding), endoprostheses collapse, other types of endoleak, and recurrent laryngeal nerve damage [3, 5]. Overall, morbidity ranges between 3 and 36%, while mortality ranges between 0 and 20% [5], and both increase with delayed surgical correction [3]. In our case series, no major complications were reported, and the procedure-related mortality was 0%.

The aim of follow-up in patients undergoing TEVAR for TTAIs is the early diagnosis of lesion progression and complications [3, 38]. However, currently, there is no clear guidance available about the most appropriate diagnostic test, timing, and duration with which to set up follow-up. In the absence of imaging abnormalities, some experts recommend repeating the examination 12–36 months later, while others suggest performing the examination every 2–5 years [3, 4]. Additional problems are the absence of long-term outcome data in the literature and the poor compliance of patients, who are often lost to follow-up. Patients are usually young and otherwise healthy, so they may consider it unnecessary to undergo diagnostic investigations. Another important aspect to be considered is that patients are afferent from a wide territory and follow-ups are hardly performed in the center where they undergo the procedure. Typically, CTA is used, but some authors prefer performing a chest X-ray associated with MRI [4, 12]. MRI is a viable alternative to CTA both in planning [39] and in patient follow-up [3, 5]: it has sensitivity and specificity in the diagnosis of aortic lesions comparable to CTA, and it has some advantages, such as better anatomical definition (including ulcers and intramural hematomas) and assessment of left ventricular function, without exposing the patient to contrast medium and radiation [3] (which could be an encouragement for young patients); cardiac evaluation is particularly important in long-term patient evaluation, as TEVAR for TTAIs has been shown to cause cardiac and aortic remodeling, that increased morbidity especially if not recognized and treated [19]. One of the problems with MRI lies in the long imaging period, which may not be tolerated by claustrophobic patients; it may also be contraindicated in patients with metal prostheses (a common condition in polytraumatized patients), clips, or pacemakers [3]. Our experience shows that MRI is a good alternative to CTA during follow-up in patients treated with TEVAR for TTAI.

The main limitations of this study lie in its design, as it is a retrospective, single-center, in the limited sample size, and in the poor follow-up.

## Conclusions

In our experience, TEVAR for TTAI has been confirmed as a safe and effective treatment. It has achieved a level of safety and efficacy that makes it the gold standard in the treatment of TTAI having excellent both short- and mid-long-term results. TEVAR is also a valid option in patients with anatomic variants. Furthermore, our experience has highlighted the feasibility of subjecting patients to MRI during follow-up to avoid the risks associated with repeated CTA throughout their lifetime, but further studies with a larger cohort of patients and a longer follow-up are required.
